# Off-harmonic optical probing of high intensity laser plasma expansion dynamics in solid density hydrogen jets

**DOI:** 10.1038/s41598-022-10797-6

**Published:** 2022-05-04

**Authors:** Constantin Bernert, Stefan Assenbaum, Florian-Emanuel Brack, Thomas E. Cowan, Chandra B. Curry, Marco Garten, Lennart Gaus, Maxence Gauthier, Sebastian Göde, Ilja Goethel, Siegfried H. Glenzer, Thomas Kluge, Stephan Kraft, Florian Kroll, Michael Kuntzsch, Josefine Metzkes-Ng, Markus Loeser, Lieselotte Obst-Huebl, Martin Rehwald, Hans-Peter Schlenvoigt, Christopher Schoenwaelder, Ulrich Schramm, Mathias Siebold, Franziska Treffert, Tim Ziegler, Karl Zeil

**Affiliations:** 1 Helmholtz-Zentrum Dresden–Rossendorf, 01328 Dresden, Germany; 2 Technische Universität Dresden, 01062 Dresden, Germany; 3 SLAC National Accelerator Laboratory, Menlo Park, CA 94025 USA; 4 University of Alberta, Edmonton, AB T6G 1H9 Canada; 5 European XFEL GmbH, 22869 Schenefeld, Germany; 6 Friedrich-Alexander Universität Erlangen-Nürnberg, 91054 Erlangen, Germany; 7 Technische Universität Darmstadt, 64289 Darmstadt, Germany; 8 Present Address: Lawrence Berkeley National Laboratory, Berkeley, CA 94720 USA

**Keywords:** Laser-produced plasmas, Plasma-based accelerators

## Abstract

Due to the non-linear nature of relativistic laser induced plasma processes, the development of laser-plasma accelerators requires precise numerical modeling. Especially high intensity laser-solid interactions are sensitive to the temporal laser rising edge and the predictive capability of simulations suffers from incomplete information on the plasma state at the onset of the relativistic interaction. Experimental diagnostics utilizing ultra-fast optical backlighters can help to ease this challenge by providing temporally resolved inside into the plasma density evolution. We present the successful implementation of an off-harmonic optical probe laser setup to investigate the interaction of a high-intensity laser at $5.4\times 10^{21}\,\hbox {W/cm}^{2}$ peak intensity with a solid-density cylindrical cryogenic hydrogen jet target of ${5}\,{\upmu }\mathrm{m}$ diameter as a target test bed. The temporal synchronization of pump and probe laser, spectral filtering and spectrally resolved data of the parasitic plasma self-emission are discussed. The probing technique mitigates detector saturation by self-emission and allowed to record a temporal scan of shadowgraphy data revealing details of the target ionization and expansion dynamics that were so far not accessible for the given laser intensity. Plasma expansion speeds of up to $(2.3 \pm 0.4)\times 10^{7}\,\hbox {m/s}$ followed by full target transparency at ${1.4}\,{\mathrm{ps}}$ after the high intensity laser peak are observed. A three dimensional particle-in-cell simulation initiated with the diagnosed target pre-expansion at ${-0.2}\,{\mathrm{ps}}$ and post processed by ray tracing simulations supports the experimental observations and demonstrates the capability of time resolved optical diagnostics to provide quantitative input and feedback to the numerical treatment within the time frame of the relativistic laser-plasma interaction.

## Introduction

Full control of high-intensity laser-matter interaction is key to enable applications like fast ignition for inertial confinement fusion^1,2^ or laser-plasma driven particle sources^3^ for warm dense matter research^4^, time-resolved studies of transient fields^5^ and translational research for radiation oncology^6^. It requires predictive power of simulation tools in quantitative agreement with experimental results and it is still challenging to achieve for laser-solid interactions^3^. One important reason is the large range of interaction physics at different time scales and laser intensities impacting the solid prior to the arrival of the laser peak^7^ and connected to that the difficulty of capturing the dynamics by a single simulation tool. Although the relativistic laser-plasma interaction can be mapped by particle-in-cell simulations^8^, the computable time window is limited and the captured physics must be dominated by the laser field. For lower intensities in the laser rising edge, approaches of radiation-hydrodynamic codes are often used to capture the longer time scales and approximate the more localized energy deposition^9,10^.

An alternative approach is the use of time-resolved experimental diagnostics that infer properties of the target shortly before the arrival of the laser peak. Microscopy techniques utilizing ultra-fast optical backlighters are a common tool to characterize the plasma density distribution in high intensity laser-solid interactions^9,11–17^. However, for ultra-relativistic laser intensities approaching $10^{22}\, \hbox {W/cm}^{2}$^18–20^, strong plasma self-emission, scattered light and surface harmonics often mask the central region of the plasma on the detector. This complicates the application of advanced probing techniques like polarimetry or few-cycle optical probing^21–23^. A promising path to overcome this issue is the shift of the probing wavelength away from the fundamental and the harmonic wavelengths of the high-intensity pump laser^24,25^, hereafter referred to as “off-harmonic optical probing”.

Here, we demonstrate this technique using a ${160}\,{\mathrm{fs}}$ backlighter at ${515}\,\mathrm{nm}$ wavelength realized by an electronically synchronized stand-alone laser system and implemented at a state of the art PW-class short pulse laser system with $5.4 \times 10^{21}\, \hbox {W/cm} ^{2}$ peak intensity and ${800}\,{\mathrm{nm}}$ central wavelength. A micrometer-sized self-replenishing jet-target of cryogenic hydrogen^26–32^ serves as a test bed and facilitates recording of optical probing data and comparison with particle-in-cell simulations. Based on spectral measurements of the plasma self-emission at different pump laser contrast conditions, the applicability of the probing technique and the importance of spectral backlighter fluence are discussed. Conducting a time delay scan, the concept enables observations of target ionization within the rising edge of the laser and target expansion before and after the peak of the pulse, so far not accessible at this intensity level. Providing realistic starting conditions for the interaction of the relativistic main pulse, a three dimensional particle-in-cell (PIC) simulation is conducted and compared to the experiment. The experimentally observed expansion dynamics are confirmed and discussed.

## Experimental setup and temporal synchonization

**Figure 1:**
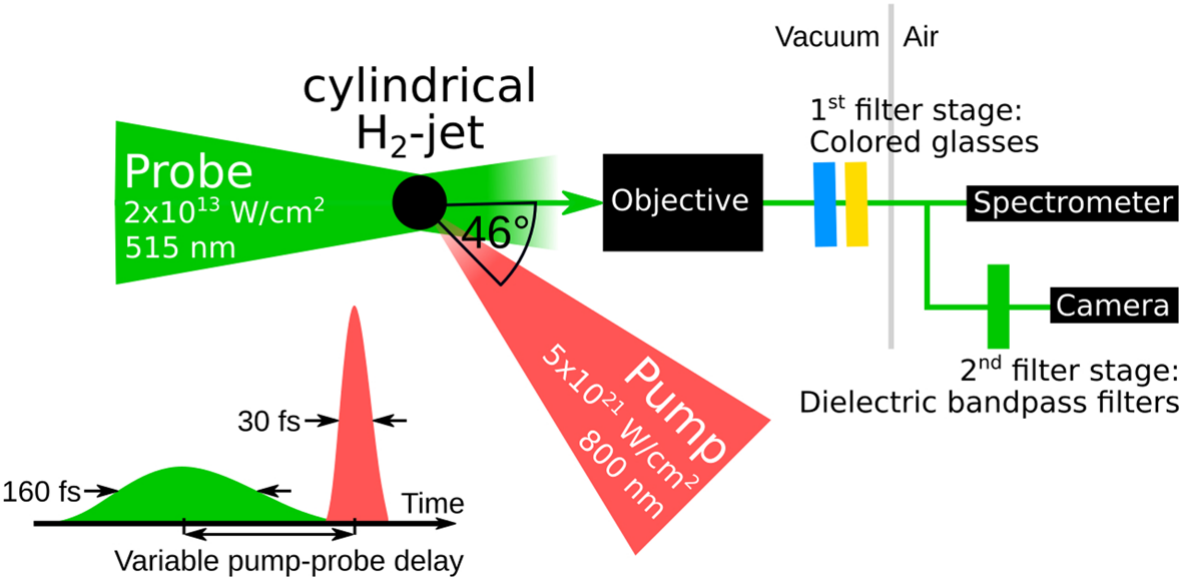
Top view of the experimental setup including a cylindrical cryogenic hydrogen jet target (black circle), pump laser (red beam), off-harmonic optical probe laser (green beam) as backlighter, imaging beamline with microscope objective (numerical aperture 0.28, working distance $80\,{\mathrm{mm}}$) and spectral filters, spectrometer and CMOS-camera for detection.

The laser-plasma experiment and the implementation of the off-harmonic optical probing system are carried out at the DRACO Petawatt laser^19^ at the Helmholtz-Zentrum Dresden–Rossendorf and the experimental setup is sketched in Fig. 1. The Titanium-Sapphire based DRACO laser (hereafter called “pump laser”, ${800}\,{\mathrm{nm}}$, ${30}\,{\mathrm{fs}}$, ${18}\,{\mathrm{J}}$ on target) is focused via an f/2.3 off-axis parabola to a spot size of ${2.6}\,{\upmu }\mathrm{m}$ full width half maximum (FWHM). The measured focal spot and the pump laser spectrum are given in the supplementary information. The resulting peak intensity is $5.4 \times 10^{21}\, \hbox {W/cm}^{2}$. High temporal contrast is achieved by applying a recollimating single plasma mirror setup^33^. In this study, a continuously flowing jet of cryogenic hydrogen^34,35^ with ${5}\,{\upmu }\mathrm{m}$ diameter serves as a solid-density target. When fully ionized it has an electron density of $5.24 \times 10^{22}\,\hbox {cm}^{-3}$, or 30 times the critical density of ${800}\,{\mathrm{nm}}$ light. Optical pulses from a secondary probe laser system^36^ (${515}\,{\mathrm{nm}}$, ${160}\,{\mathrm{fs}}$) are used as off-harmonic backlighter at an angle of $46^\circ$ to the pump laser. The shadow of the target is imaged by a finite conjugate long working distance objective with a magnification of 30.5 onto a camera outside the vacuum chamber (for details see Methods section). The spatial resolution limit is measured to be ${1.5}\,{\upmu }\mathrm{m}$ and movements in the time window of the laser pulse duration are blurred in the observation.

For long-term availability of optical probing capabilities of a laser-plasma laboratory it is beneficial to implement an independent laser system instead of picking a probe from the pump laser beam after compression. The alignment of target and probe is completely independent from the pump laser settings and by utilizing different laser media for pump and probe laser the possibility for off-harmonic probing is intrinsically given. An important aspect is the temporal synchronization of both laser systems and two options are commonly used. Either pump and probe laser share the same laser oscillator^24^ or a stand-alone probe laser oscillator is electronically synchronized to the pump laser oscillator^25^. Here, the performance of the second option by utilizing commercially available lock-electronics together with one centralized clock for both lasers is presented (for details see Methods section). In general, the temporal synchronization is based on three pillars. The first is the high-frequency synchronization at the level of the laser oscillator frequency or higher. It determines the temporal shot-to-shot stability of the pump-probe delay in the experiment and is desired to be close to the laser pulse duration. The core is a highly stable external clock laser. The optical signal of this laser is distributed over individual fiber links to the lock-electronics of pump and probe laser oscillator, each located in a different experimental cave. Accumulating the electronic and optical jitters in the whole chain up to experiment yields an RMS pump-probe delay stability of $230\,{\mathrm{fs}}$. The second pillar is the low-frequency synchronization on the level of the laser-amplifier repetition rate and the third pillar is the active synchronization control via a beam arrival monitor (BAM). The BAM is a tool that allows for on-shot measurement of the timing between both amplified laser pulses. It is realized by splitting off a part of each laser pulse and overlapping them in space and time by a well characterized measurement process, e.g. self-guiding and probing of the resulting filament in air. The timing uncertainty of a BAM measurement is ${50}\,{\mathrm{fs}}$^25^. Together with the pulse duration of the probe laser, the temporal resolution of a time delay scan is calculated to be $175\,{\mathrm{fs}}$.

For the present installation, the temporal synchronization stability of the pump-probe setup is sufficiently precise to scan the high-intensity laser-matter interaction within hundreds of fs around the pump laser pulse.

## Plasma emission and spectral filtering

**Figure 2:**
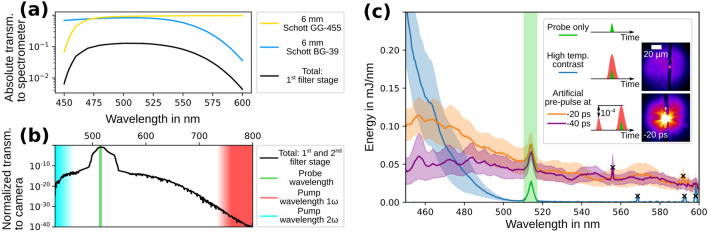
**(a)** Calculated absolute spectral transmission function from the target up to the spectrometer (refer Fig. 1) together with the individual transmission of the two colored glass filters; **(b)** Normalized transmission function up to the camera; **(c)** Spectral measurement of the plasma self-emission for three different laser contrast settings: High temporal contrast (blue) and artificial pre-pulse at ${-20}\,{\mathrm{ps}}$ (orange) and ${-40}\,{\mathrm{ps}}$ (violet) before the pump pulse. Each setting shows the mean over consecutive shots and the corresponding standard error of the mean as shaded area, the probe wavelength is marked by a green shaded area. Parasitic gamma-ray signals on the detector are marked by black crosses. Exemplary shadowgrams for high contrast and ${-20}\,{\mathrm{ps}}$ pre-pulse setting in the legend.

Equally important to the appropriate choice of the backlighter wavelength is the discrimination of the probe signal from the plasma self-emission. Both sufficient spectral filtering and spectral fluence of the probe are needed. Dielectric coatings on vacuum windows and spectral bandpass filters can have low damage thresholds and need to be protected against high fluences, especially from the ultraviolet light of the $3^{\mathrm{rd}}$ and $4^{\mathrm{th}}$ harmonic of the pump laser. This is solved by a two-stage approach of spectral filtering (for details see Methods section). The first filter stage is placed directly behind the objective and the second filter stage in front of the camera (refer to Fig. 1). The calculated transmission function of the imaging beamline from the manufacturers data of all components up to the spectrometer (only first filter stage) and up to the camera (both filter stages) is presented in Fig. 2a,b. In Fig. 2b the spectral band of the probe (${515}\,{\mathrm{nm}}$) is shown in green, the band of the pump in red ($1 \omega$) and the band of the second harmonic of the pump ($2 \omega$) in turquoise. The calculation yields that the transmission ratio between the probe spectrum and the spectral band of the fundamental (second harmonic) of the pump up to the camera is better than $10^{32}$ ($10^{15}$). The calculation does not take into account transient effects like saturated absorption in the colored glass. Thus, within the strongly absorbing bands of the glasses, the attenuation may be lower.

The efficiency of the first filter stage against the fundamental and the harmonics of the pump laser is measured with the spectrometer in the spectral band between ${200}\,{\mathrm{nm}}$ to ${1100}\,{\mathrm{nm}}$ (view spectrometer position in Fig. 1). Independent of the pump laser setting, no signal is measured outside the spectral band from ${450}\,{\mathrm{nm}}$ to ${600}\,{\mathrm{nm}}$. This is due to the strong filter effect of the glasses. The correction of the spectrometer signal by the transmission function of the first filter stage (Fig.. 2a) enables a spectral characterization of the plasma self-emission between ${450}\,{\mathrm{nm}}$ and ${600}\,{\mathrm{nm}}$. The results are displayed in Fig. 2c for different laser contrast conditions. Each graph shows the mean over consecutive shots and the corresponding standard error of the mean as shaded area. For each setting the variation of the spectra is mainly due to slight variations in the pump laser target overlap. The spectra are calibrated to an absolute scale in mJ/nm by the known amount of energy in the probe spectrum (highlighted in green) and reflect the emitted spectral energy into the numerical aperture of the objective. The blue graph represents the spectrum for the “high temporal contrast” setting and shows the tail of the second harmonic of the pump laser. To emulate a “low contrast setting”, an artificial pre-pulse at ${-20}\,{\mathrm{ps}}$ or ${-40}\,{\mathrm{ps}}$ is inserted (orange and violet curve). The pre-pulse is generated via an optical shortcut over a small pick-off mirror in the pump laser beamline (see supplementary information). It has an intensity four orders of magnitude lower than the pump pulse. Both spectra show that the tail of the second harmonic vanishes and a close to homogeneous spectral emission between ${450}\,{\mathrm{nm}}$ and ${600}\,{\mathrm{nm}}$ emerges. The different plasma emission characteristics can be explained as follows. Highest conversion efficiency for second harmonic generation on solid density targets is achieved for plasma density surface-scalelengths shorter than the pump laser wavelength^37–39^. Depending on the pre-pulse timing, the pre-pulses utilized here generate a scalelength of multiple pump laser wavelengths. Therefore, the observation of a reduced signal of the tail of the second harmonic with increased pre-pulse delay is expected. The homogeneous spectral emission that is observed for both pre-pulse settings can be attributed to an interplay between several mechanisms. First, the generation of a plasma density surface-scalelength increases the coupling efficiency of laser energy into electrons and produces a higher number of photons in a broad energy range^9,40,41^. Second, as the target is pre-expanded it features a larger surface area to emit thermal radiation. Third, the increased amount of undercritical plasma acts as a highly non-linear medium to the incident pump laser and spectral broadening and self-phase modulation occur^42–44^.

The technical implication of the measured plasma self-emission to off-harmonic optical probing becomes clear by comparing the signal heights of the self-emission to the probe spectrum (probe only) in Fig. 2c. As the second filter stage in front of the camera has an optical density $\ge {10}$, only the amount of light in the spectral band of the probe needs to be considered for estimating the contrast between probe signal and plasma self-emission on the camera. The probe spectrum is visible as additive spectral energy for all spectra on top of the self-emission. Comparing the spectral energy in the probe spectrum with the amount of spectral energy in the plasma self-emission for the same wavelengths, it can be deduced that the probe energy is too low to outshine the plasma for both pre-pulse contrast settings. To quantitatively estimate the required amount of probe energy to outshine the plasma an additional spatial factor needs to be taken into account. The plasma self-emission mostly originates from a narrow point while the probe beam illuminates a larger field of view. This is illustrated in two exemplary shadowgrams in the legend of Fig. 2c. By adding defined attenuation with filters on the camera we measured that the shadowgram of the pre-pulse contrast setting would require a factor 100 higher probe pulse energy to restrict the self-emission to the same spatial extent like in the high contrast setting. That is a factor of $\sim {20}$ higher than one would estimate solely from the spectra and reflects that the probed field of view is about five times larger in diameter than the plasma self-emission. As spectral fluence scales with the square of the beam diameter, a clever choice of diameter is key to find a good compromise between plasma self-emission and field of view.

In general, viewing the non-spatial parameters of the probe beam, e.g. pulse duration and energy, it is important to consider that the Fourier-limited laser pulse duration is inherently linked to spectral bandwidth by the time-bandwidth product. Shorter probe pulses require a broader bandwidth and the pulse energy is always distributed over the whole spectrum. It follows that, at constant pulse energy, shorter pulses have a lower spectral energy per unit wavelength than longer pulses. However, for a probing image without saturation of the detector it is required that the probe beam is spectrally brighter than the plasma self-emission but at the same time the probe needs to stay non-invasive, i.e. non-ionizing. Depending on the pump laser contrast, a concentration of the probe pulse energy into a narrow bandwidth can be beneficial. For example, reducing the time resolution by using a picosecond probe in conjunction with narrow bandpass filters on the camera could outshine the self-emission in the ${-40}\,{\mathrm{ps}}$ pre-pulse setting and at the same time keep the probe intensity below the ionization threshold of the target. In contrast to that, very short and broadband probe pulses require at least a part of the spectral bandwidth to be brighter than the plasma self-emission. This is possible in the high-contrast setting of the pump laser. Here the off-harmonic optical probing technique could evolve to even higher temporal resolution than presented here and as it is already shown for a lower pump laser intensity of $4 \times 10^{19}\, \hbox {W/cm}^{2}$^23^. For few-cycle probe pulses it has been shown that spectral filtering behind the probed interaction does not diminish the time resolution of the probing data^22^. A careful choice of spectral filters and a precise characterization of the plasma self-emission will enable future developments into this direction.

## Optical shadowgraphy results

**Figure 3:**
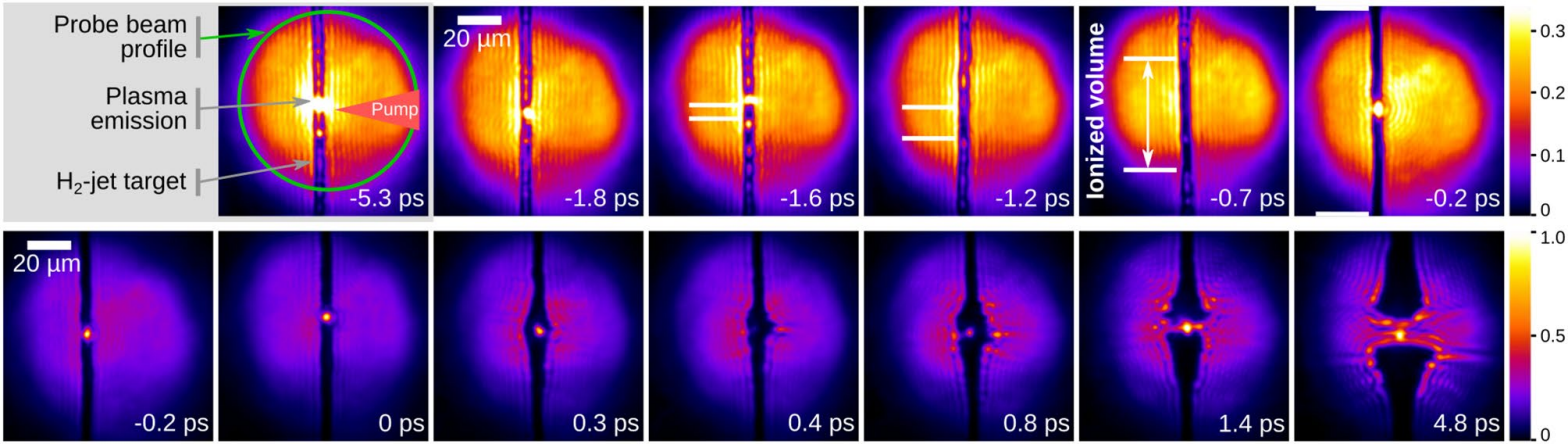
Shadowgraphy images for different pump-probe delays at $5.4 \times 10^{21}\, \hbox {W/cm}^{2}$ pump laser intensity. The temporal resolution of the time delay scan is ${175}\,{\mathrm{fs}}$. The pump and probe laser settings are equivalent for all images. For better visibility of the onset of target ionization (indicated between the horizontal white bars) and the subsequent plasma expansion dynamics, the color scale is different in both rows.

The appropriate choice of backlighter wavelength, sufficient spectral filtering in the imaging beamline and a good contrast between spectral probe laser fluence and spectral plasma self-emission enables the optical investigation of the plasma dynamics at $5.4 \times 10^{21}\, \hbox {W/cm}^{2}$ pump laser intensity, e.g. via shadowgraphy probing. Figure 3 shows a collection of shadowgrams of the interaction of the high intensity pump laser with the cylindrical cryogenic hydrogen jet target. For each shadowgram the individual pump-probe delay is given in the lower right corner. The absolute timing between different shots is obtained by the BAM (see Methods section). An example of the generally visible features of each shadowgram is given in the upper left of Fig. 3. Residual plasma self-emission occurs occasionally and it is limited to a small spot. For better visualization of the onset of target ionization and the subsequent dynamics the color scales in the upper and lower rows of the figure are different (continuity is given at ${-0.2}\,{\mathrm{ps}}$).

For all shadowgrams that are captured earlier than ${-1.8}\,{\mathrm{ps}}$ the hydrogen target is transparent throughout the whole field of view (see transmitted probe light along the entire target). The target acts like a cylindrical lens and rays that are close to the edge of the target get refracted outside the objective. Exept for the residual plasma self-emission in the center, the shadowgrams are equivalent to the images where no pump laser is interacting with the target. At ${-1.6}\,{\mathrm{ps}}$ the target shows a small darkened volume at the position where the pump laser hits the target. The spatial extent of the darkened volume grows with increased pump-probe delay. For a better visualization, the dark volume is highlighted by two horizontal white bars in all shadowgrams between ${-1.6}\,{\mathrm{ps}}$ and ${-0.2}\,{\mathrm{ps}}$. The darkening indicates the phase transition of parts of the target from the initially solid dielectric state^45^ into a partially ionized plasma, where the density of electrons in the conduction band is close to or higher than the critical plasma density of the probe wavelength ($n_c[{515}\,{\mathrm{nm}}] = 4.21 \times 10^{21}\, \hbox {cm}^{-3}$).

In the rising edge of the pump laser, where the intensity is well below the relativistic limit ($\sim 10^{18}\, \hbox {W/cm}^{2}$) and close to the intensity of laser induced dielectric breakdown ($< 10^{14}\, \hbox {W/cm}^{2}$), the creation of “free” electrons is explained by strong field ionization and avalanche ionization between bound electrons and laser-heated conduction band electrons^46^. Depending on the instantaneous pump laser intensity the optical ionization process can be described as a mixture of multi-photon- and tunnel-ionization, specified in the Keldysh picture^47,48^. Due to the complex valued refractive index of the created mixture of plasma and solid, the traversing probe light is either absorbed, reflected or refracted outside the aperture of the objective. From the shadowgraphy data alone, it cannot be distinguished whether the plasma is present only on the cylindrical surface or in the target bulk.

The onset of target ionization has already been used for temporally resolved intensity contouring (TRIC) of high-intensity laser pulses on thin plastic targets up to a pump laser peak intensity of $6 \times 10^{19}\, \hbox {W/cm}^{2}$^49^. Following the arguments of this technique and assuming barrier-suppression-ionization as threshold intensity $I_{th}$ of ionization^49^, it can be estimated that the pump laser is surpassing $1.4\times 10^{14}\, \hbox {W/cm}^{2}$ between ${-1.8}\,{\mathrm{ps}}$ and ${-1.6}\,{\mathrm{ps}}$. However, the cylindrical target shape is non-ideal for the implementation of TRIC and as mentioned, the ionization mechanisms are more diverse. It is possible that the target front side shows signs of ionization even earlier than they are observable in this geometry. Additionally, the focussing shape of the target complicates the spatial allocation of the onset of ionization.

The time-dependent increase of the opaque target volume between ${-1.6}\,{\mathrm{ps}}$ and ${-0.2}\,{\mathrm{ps}}$ is identified as a complex interplay between two sources. First, the spatial extent of the pump laser focus in combination with the temporally rising intensity on target (see TRIC^49^) and second by the dissipation of hot electrons from the center of the interaction when the pump laser intensity becomes relativistic. The hot electrons stream into the surrounding target volume and cause additional colder return currents. Thus, the hot electrons ionize the solidified hydrogen either directly by collisions or indirectly by field-ionization^50,51^.

For pump-probe delays later than ${-0.2}\,{\mathrm{ps}}$ (lower row in Fig. 3) the target is ionized throughout the whole field of view (${0.1}\,{\mathrm{mm}} \times {0.1}\,{\mathrm{mm}}$). While the target shape at the position of the pump laser focus is generally unchanged at ${-0.2}\,{\mathrm{ps}}$ and ${0}\,{\mathrm{ps}}$ (possible small-scale changes are hidden by plasma self-emission), the target shows an expansion of the plasma for delays between ${0.1}\,{\mathrm{ps}}$ and ${0.8}\,{\mathrm{ps}}$. For even later pump-probe-delays ( $> {1.4}\,{\mathrm{ps}}$) the plasma has a more symmetric shape and the probe laser is able to penetrate the whole central volume of the plasma, i.e. the target is fully transparent.

## Discussion of the plasma expansion dynamics

**Figure 4:**
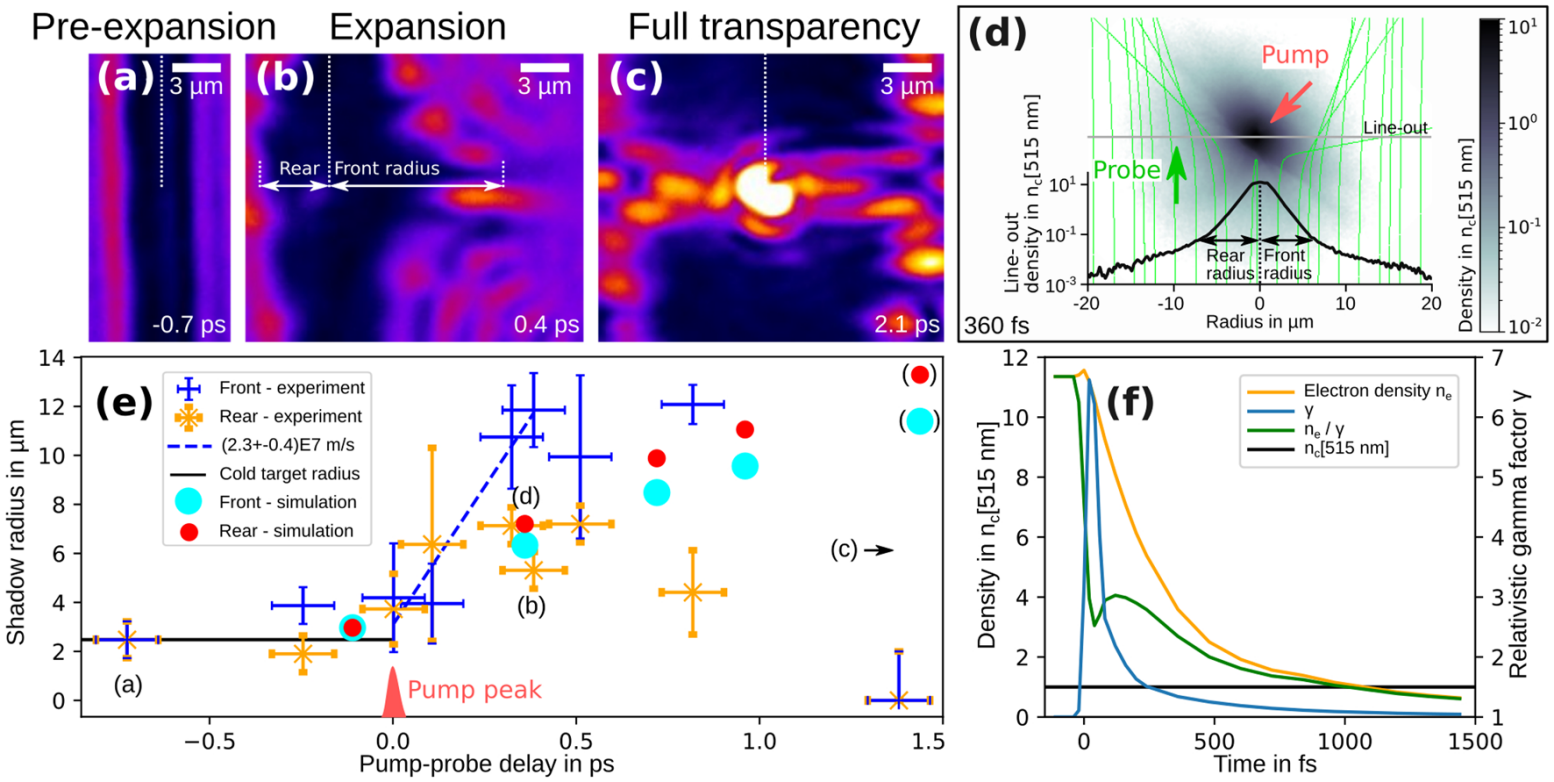
Close up of three shadowgraphy images, each representative for three characteristic transient target states: **(a)** target ionization and pre-expansion by the pump laser rising edge; **(b)** rapid plasma expansion after the pump laser peak; **(c)** full transparency of the target after single picoseconds. The color scale is consistent in all images. **(d)** Ray tracing of the PIC-simulation results: Top view slice of the density $n_e / \gamma$ at ${360}\,{\mathrm{fs}}$ after the pump laser peak is shown in gray color scale. The refractive index is calculated by formula 1 and inserted into the object plane of an imaging setup in Zemax with similar imaging properties like the experimental setup. The bending of the probe rays in the object plane is visualized by the green lines. The simulated front and rear side shadow radius are retrieved in the image plane and presented together with a horizontal line-out of the density. The spatial scale of the line-out and the top view slice is equivalent. **(e)** Evolution of the shadow radii for different pump-probe delays in the experiment (crosses) and ray tracing of the PIC simulation result (circles, brackets indicate the occurrence of transmitted light within the shadow). **(f)** Temporal evolution of $n_e$, $\gamma$ and $n_e / \gamma$ from the PIC simulation averaged over the initial target bulk (${2.5}\,{\upmu }\mathrm{m}$ radius and ${1}\,{\upmu }\mathrm{m}$ along the target axis).

Three characteristic transient target states are identified, each in a different time domain. Close-ups of representative shadowgrams are depicted in Fig. 4a–c. Figure 4a shows the target ionization and pre-expansion by the pump laser’s rising edge, Fig. 4b shows the fast expansion of the target after the pump laser peak and Fig. 4c shows the full transparency of the target. As sketched in Fig. 4b, a front and rear side shadow radius can be measured from each shadowgram. The central axis of the target is determined by the undisturbed outer parts of the interaction. While the shadow at the front side shows prominent spikes, the shadow at the rear side is spatially more homogeneous. Thus the measured rear side radius reflects the more homogeneous bulky expansion, whereas the front side radius shows the extend of the largest observed spike. In Fig. 4e the front (rear) radii are plotted as blue (orange) crosses versus pump-probe delay. The spatial errorbars originate from the limited spatial resolution, the reduced observational accuracy by occasional plasma emission or a faint transition of the shadow into the signal level of the probe illumination. Between ${-1.6}\,{\mathrm{ps}}$ and ${-0.7}\,{\mathrm{ps}}$ there is no deviation of the plasma radii from the cold target diameter. Within the error bars the same holds true for ${-0.2}\,{\mathrm{ps}}$ and ${0}\,{\mathrm{ps}}$, although a slight trend of pre-expansion is visible. The arrival of the pump laser peak causes a rapid growth of the shadow radii, with the front radius increasing to higher values than the rear radius. The maximum velocity of the shadow expansion is observed at the front side and fitted to be $(2.3\pm 0.4)\times 10^{7}\, \hbox {m/s}$ (dashed blue line) between ${0}\,{\mathrm{ps}}$ and ${0.4}\,{\mathrm{ps}}$. Subsequently the growth of the shadow radii first stagnates and then reverses until full transparency of the target is reached at ${1.4}\,{\mathrm{ps}}$.

The ultra-fast plasma expansion following the pump laser peak occurs on a time-scale close to the probe laser pulse duration and motional blurring of the individual shadowgrams is expected. To compare the experimental obervation to the theoretically expected plasma expansion dynamics and to get more inside into the role of relativistically induced transparency to full target transparency at ${1.4}\,{\mathrm{ps}}$, a three dimensional PIC simulation with the code PIConGPU^52,53^ version 0.4.3 is conducted (see Methods section). In the PIC simulation the target is initialized as a fully ionized plasma cylinder with a density of solid hydrogen and a diameter of ${5}\,{\upmu }\mathrm{m}$. An additional exponential surface density scalelength $L_p$ is introduced to model the experimental observation of a slightly increased target diameter to $(5.77\pm 0.75)\,{\upmu }\mathrm{m}$ at ${-0.2}\,{\mathrm{ps}}$. To find the best fitting scalelength, ray-tracing simulations of the plasma cylinder with various surface scale length are conducted. Given the experimental error bars the best fitting $L_p$ measures between ${0.06}\,{\upmu }\mathrm{m}$ and ${0.13}\,{\upmu }\mathrm{m}$. $L_p = {0.1}\,{\upmu }\mathrm{m}$ is initialized for the plasma cylinder in the simulation. The time window of the simulation starts at ${-110}\,{\mathrm{fs}}$ and stops at ${1440}\,{\mathrm{fs}}$ after the pump laser peak reaches the target center.

Due to the mass ratio of protons and electrons, light transparency of plasmas is governed by electron interaction. For relativistic laser plasmas, the high velocity of electrons can be considered as a mass increase quantified by the relativistic gamma factor $\gamma$^54^. Therefore, a top view slice of the electron density $n_e$ divided by the local mean of $\gamma$ from the PIC simulation is shown in gray color scale in Fig. 4d. Because the experimentally measured shadow expansion is maximized at ${0.4}\,{\mathrm{ps}}$, the top view slice shows $n_e / \gamma$ at ${360}\,{\mathrm{fs}}$. It is retrieved by averaging the particles in the three dimensional simulation box within ${1}\,{\upmu }\mathrm{m}$ along the target axis in the plane of the pump laser maximum. The pump-probe geometry is indicated by the red and green arrow. A lineout of the density $n_e / \gamma$ along the central horizontal axis of the top view slice is shown at the bottom. The values of the line-out are averaged over ${1}\,{\upmu }\mathrm{m}$ along the vertical axis of the top view.

To compare the PIC simulation to the experiment, the shadowgram formation for a given particle density distribution obtained from the PIC simulation needs to be modeled. For this, a dispersion relation of the plasma is required and the refractive index ${\tilde{n}}$ is commonly calculated from $n_e / \gamma$ via the formula^54^

1 $$\begin{aligned} {\tilde{n}} = \sqrt{1 - \frac{n_e}{\gamma \cdot n_c}} \end{aligned}$$

with $n_c$ being the critical density of the probe wavelength. The spatial distribution of the refractive index is inserted into the object plane of an imaging setup in Zemax (Zemax 13 Release 2 SP6 Professional (64-bit)) with similar imaging properties like the experimental setup. The bending of the probe rays penetrating the strongly refracting plasma distribution is shown as green lines together with the top view slice in Fig. 4d. The ray tracing allows to retrieve simulated front and rear side shadow radii in the image plane. For the particle density at ${360}\,{\mathrm{fs}}$, the retrieved shadow radii are indicated by horizontal arrows in the density line-out at the bottom of the figure. Probe rays, that are captured by the opening angle of the objective and by this contribute to the shadowgram formation, propagate through the plasma in such a way, that they do not penetrate densities higher than $0.1 \, n_c$. This illustrates that the shadow formation is governed by refraction on the density gradients of the plasma and that the dispersive properties of the plasma are of specific importance here.

The simulated front and rear side shadow radii from the particle densities at ${-110}\,{\mathrm{fs}}$, ${360}\,{\mathrm{fs}}$, ${720}\,{\mathrm{fs}}$, ${960}\,{\mathrm{fs}}$ and ${1440}\,{\mathrm{fs}}$ are plotted together with the experimental data in Fig. 4e as circular markers. Comparing the simulated front and rear side radius at ${360}\,{\mathrm{fs}}$ with the experimental data, the rear side shadow radius is well matched by the simulation. However, the radius at the front side is underestimated. A possible reason could be the difference in spatial shape of the shadowgrams at the front and at the rear side in the experiment. While the front side shows a more structured shadow contour with several spikes and the front radii reflect the extend of the largest spike, the rear side radii are measured from a spatially more homogeneous expansion. For the ray tracing a spatial average over particle density of the PIC simulation (top view slice in Fig. 4d) is used to calculate the refractive index distribution. It follows, that the simulated shadow radii represents the expansion of the target bulk rather than small scale structures. This explains the better congruence of experiment and simulation results for the rear side radius. The simulated front side radius at ${360}\,{\mathrm{fs}}$ reasonably compares to the experimental observation when the spikes in the experimental shadowgram would be neglected.

For investigating the origin of the spikes, a more detailed view into the simulation results would be required. The same applies for the plasma dynamics for times between ${-110}\,{\mathrm{fs}}$ and $\sim {360}\, {\mathrm{fs}}$, which is outside the experimental capabilities of the current probe setup. However, for this a more careful treatment of the dispersion relation of the plasma is needed. The pump laser induces a high non-equilibrium state of the plasma. The laser heats electrons and by this induces strong charge separation fields that subsequently dominate the physics of the plasma system. Protons and electrons are spatially non-isotropically distributed and both species expand at high speed into the surrounding vacuum. Usual approximations for the derivation of the dispersion relation, like negligence of the temporal variation of ionic currents as well as spatial gradients in $n_e$ and the proton density $n_p$, have to be tackled and the usage of formula 1 becomes questionable. A rigorous treatment requires a dedicated solution of the plasma wave equation for the propagation of the probe light. A possible future approach for simulating the shadow formation will be the propagation of the probe light within the PIC simulation, where Maxell’s equations are solved explicitly. Because of the comparably long probe laser pulse duration of ${160}\,{\mathrm{fs}}$ in the presented experiment and the challenging interpretation of small scale structures especially at time scales close to the pump laser peak, we here restrict the presentation of simulation results to the time steps ${-110}\,{\mathrm{fs}}$, ${360}\,{\mathrm{fs}}$, ${720}\,{\mathrm{fs}}$, 960 fs and ${1440}\,{\mathrm{fs}}$, where $n_e$, $n_e / \gamma$ and $n_p$ are spatially nearly equally distributed.

Another experimental observation is the full transparency of the target at ${1.4}\,{\mathrm{ps}}$. Although plasma self-emission masks central regions of the shadowgram and the temporal resolution of the time delay scan is ${175}\,{\mathrm{fs}}$, the penetration of probe light through central regions of the target demonstrates, that $n_e / \gamma$ must have dropped below $n_c$ along the complete propagation for this time scale. The PIC simulation can be used as a guide to disentangle the contribution of relativistically induced transparency (RIT) of the target for the probe light and the density reduction by plasma expansion into vacuum. Figure 4f shows the temporal evolution of $n_e$ (orange), $\gamma$ (blue) and $n_e / \gamma$ (green). All quantities are volumetric averages over the initial target bulk of ${2.5}\,{\upmu }\mathrm{m}$ radius and ${1}\,{\upmu }\mathrm{m}$ along the target axis. The evolution of $\gamma$ shows, that significant relativistically induced density reduction is mainly present in the first ${200}\,{\mathrm{fs}}$ after the pump laser peak. The fast increase of $\gamma$ lowers the average $n_e / \gamma$ down to $3 \, n_c$ at ${40}\,{\mathrm{fs}}$. It follows that for the discussed target geometry, material and pump laser intensity the heating is not sufficient to induce full target transparency for the probe by RIT right after the pump pulse. For time scales $> {200}\,{\mathrm{fs}}$, $\gamma$ converges towards unity and $n_e / \gamma$ converges to $n_e$. The evolution of $n_e$ shows the effect of density reduction by plasma expansion into the surrounding vacuum. As expected, $n_e$ drops over time. At $\sim {1070}\,{\mathrm{fs}}$ $n_e$ has fallen below $n_c$ and at ${1440}\,{\mathrm{fs}} \,n_e$ has reached ${0.64} \, n_c$. Although the whole target has under-critical density at this time, the gradients of the refractive index are still high and a shadowgram is retrieved by ray tracing (see radii from simulation in Fig. 4e at ${1.44}\,{\mathrm{ps}}$). The majority of rays close to the central axis is refracted to angles larger than the opening angle of the objective, but in contrast to the simulated shadowgrams at shorter times a low but significant amount of transmitted light at the percent level is observed. The simulated shadow radii are therefore given in brackets. Note, that especially at these long time scales the absorbing boundary conditions of the PIC simulation can influence the temporal evolution of the plasma expansion. Electrons with $n_e > 10^{-3} \, n_c$ and protons with $n_p > 10^{-3} \, n_c$ reach the bounds of the simulation box already at ${60}\,{\mathrm{fs}}$ and ${360}\,{\mathrm{fs}}$, respectively. This can cause differences between simulated and experimentally measured shadow radii at later times. A possible improvement of the simulation could be subsequent modeling of the plasma expansion by a two-temperature hydrodynamics simulation with a well defined interconnection point to the PIC simulation at hundreds of femtoseconds after the pump laser peak.

The temporal evolution of $n_e$ and $\gamma$ in the PIC simulation indicate, that the experimentally observed full transparency of the target at ${1.4}\,{\mathrm{ps}}$ is most likely not caused by RIT but results from the reduction of the central plasma density by plasma expansion into vacuum.

## Conclusion

We demonstrated the application of an off-harmonic optical probing setup at a high intensity laser interaction with a cylindrical cryogenic hydrogen jet target. We have shown the temporal synchronization concept and confirmed the necessity of a beam arrival monitor if a timing precision comparable to or below the probe laser pulse duration is required. We explored the laser-contrast-dependent spectral emission characteristics of the plasma self-emission and discussed the results and its implications on optical probing of similar interactions concerning the choice of backlighter wavelength, spectral bandwidth, spectral fluence and spectral filters. The efforts and concept allowed to make hitherto impossible observations of the target evolution via optical shadowgraphy at a pump laser intensity of $5.4 \times 10^{21}\,\hbox {W/cm}^{2}$. Three successive characteristic target states are identified: First ionization together with target pre-expansion by the pump laser’s rising edge, the fast expansion of the target after the pump laser peak and full transparency of the target after single picoseconds . The onset of target ionization is observed between ${-1.8}\,\mathrm{ps}$ and ${-1.6}\,\mathrm{ps}$ and the measurement of a target diameter of $(5.77\pm 0.75)\,{\upmu }\mathrm{m}$ instead of nominally ${5}\,{\upmu }\mathrm{m}$ at $(-0.2 \pm 0.1)\,\mathrm{ps}$ allowed to restrict the target pre-expansion to a plasma density surface scalelength between ${0.06}\,{\upmu }\mathrm{m}$ and ${0.13}\,{\upmu }\mathrm{m}$ . This provided realistic starting conditions for three dimensional particle-in-cell simulations of the ultra-relativistic laser plasma interaction and bridged the necessity of simulating the non-relativistic laser rising edge. A quantitative comparison of the experimentally observed fast plasma expansion with speeds of up to $(2.3 \pm 0.4)\times 10^{7}\,\hbox {m/s}$ to the particle-in-cell simulation results was enabled by utilizing ray tracing of the simulated plasma density evolution. It was shown that shadow formation in the experimental diagnostic is governed by refraction at the plasma density gradients. Full target transparency is experimentally observed after $(1.4 \pm 0.1)\,\mathrm{ps}$. For that time the simulation suggests, that the reduction of the central plasma density is governed by plasma expansion into vacuum and the contribution of relativistically induced transparency is negligible.

In conclusion, we have shown that off-harmonic optical shadowgraphy probing provides realistic initial target parameters as input for three dimensional PIC simulations at the discussed laser intensities and the technique is able to provide quantitative feedback to simulations of the relativistic laser-plasma interaction.

## Methods

### Probe laser and microscopy beamline

The probe laser pulses with ${100}\,{{\upmu } \mathrm{J}}$ energy, ${515}\,{\mathrm{nm}}$ central wavelength, ${3}\,{\mathrm{nm}}$ spectral FWHM (spectrum in supplementary information) and ${160}\,\mathrm{fs}$ pulse duration are focused to a field of view of ${60}\,{\upmu }\mathrm{m}$ FWHM. This yields a fluence of ${4}\,\hbox {J/cm}^{2}$ and an intensity of $2\times 10^{13}\, \hbox {W/cm}^{2}$ in the target plane. The probe laser pulses are generated via second harmonic generation from laser pulses of an Ytterbium based laser system. It consists of a commercially available laser oscillator (“FLINT” by LightConversion) and a regenerative amplifier using chirped pulse amplification^36^. The imaging system of the target uses a custom-made finite conjugate apochromatic objective with a numerical aperture (NA) of 0.28 and a working distance of ${80}\,{\mathrm{mm}}$. The imaging beamline utilizes Aluminum-mirrors and a fused-silica window with anti-reflection coating for the probe wavelength as well as colored glass-filters. To be recorded with two different diagnostics, the image is split by a non-polarizing beamsplitter cube. One image is captured on a cosine-corrector attached to an intensity calibrated optical spectrometer. The other image is recorded by a camera (Allied Vision Prosilica GT1600, ${4.4}\,{\upmu }\mathrm{m}$ pixel size, ${1600} \times {1200}$ pixels), that is equipped with two dielectric bandpass filters. The overall field of view (FOV) on the camera is ${200}\,{\upmu }\mathrm{m} \times {180}\,{\upmu }\mathrm{m}$, while only ${100}\,{\upmu }\mathrm{m} \times {100}\,{\upmu }\mathrm{m}$ FOV are presented in the manuscript (Fig. 3). By imaging a resolution test chart, no image distortions are found within the overall FOV and the spatial resolution limit is measured to be ${1.5}\,{\upmu }\mathrm{m}$ (see supplementary figure S2a,b). No change in imaging quality is observed within ${10}\,{\upmu }\mathrm{m}$ longitudinal movement of the test chart.

### Temporal synchronization

A schematic of the temporal synchronization setup is given in the supplementary Fig. S1. The optical master oscillator (OMO) is distributed to the individual oscillators over actively stabilized fiber links to compensate drift (e.g. thermal, vibrations). The stabilization of the fiber length is realized by a feedback loop which is fed by a balanced cross-correlator signal between the OMO pulses and partially back-reflected laser pulses from the end of the fiber link. The temporal synchronization of the pump and probe laser is a commercially available solutions from Menlo Systems that consists of balanced optical to microwave phase detector (BOM-PD), phase-shifter (DDS 120), mixer-detector-unit (MDU) and a lock electronic (RRE Syncro). The BOM-PD converts the OMO laser pulses into an RF-signal with very low timing jitter. It corresponds to the 25th harmonic (${1950}\,{\mathrm{MHz}}$) of the fundamental repetition rate of ${78}\,{\mathrm{MHz}}$. The oscillators are locked at the 26th harmonic that is detected via an MDU. The differential frequency between the BOM-PD and the 26th harmonic is given on a phase detector with ${78}\,{\mathrm{MHz}}$ from the DDS120. The error signal of the phase lock loop is used to adapt the cavity length of the oscillator by changing the voltage of a piezo stage. This minimizes the temporal jitter between the OMO and the oscillators of the pump and the probe laser system. The DDS 120 can be used as a phaseshifter in the synchronization setup to change the phase between the OMO and the oscillator. It enables software-controlled sub ${10}\,\mathrm{fs}$ time delay steps and thus overcomes the necessity of optical delay stages in the experimental area.

To benchmark the temporal delay stability between pump and probe laser we first conduct noise measurements of each individual laser oscillator with a stable external clock (FSWP by Rohde-Schwarz) and secondly of the combined and amplified laser systems on the beam arrival monitor (BAM)^25^. The supplementary Fig. S1c,d present the single sideband phasenoise (solid gray - free running and solid blue - synchronized) and the calculated integrated squared RMS jitter (dashed blue line) of both oscillators. The covered frequency range of the piezo stage in each oscillator is seen in the reduction of phase noise up to a frequency of $\sim {1}\,{\mathrm{kHz}}$. The temporal integrated RMS jitter between ${1}\,{\mathrm{Hz}}$ and ${20}\,{\mathrm{kHz}}$ is measured to be ${119}\,\mathrm{fs}$ for the pump and ${152}\,\mathrm{fs}$ for the probe oscillator. The overall system performance after all optical amplifiers can be seen in the supplementary Fig. S1b as the result of a BAM-measurement for 150 consecutive shots at ${1}\,{\mathrm{Hz}}$. As reported previously, the time delay between pump and probe laser for each shot can be determined retrospectively with ${50}\,\mathrm{fs}$ accuracy^25^. From this data we retrieve an RMS pump-probe delay stability of ${230}\,\mathrm{fs}$. The quadratic subtraction of the measured integrated RMS jitter of each oscillator from this value yields ${125}\,\mathrm{fs}$ residual delay jitter that originates from the optical path length between the two oscillators and the experiment ($\sim {0.5}\,{\mathrm{km}}$).

### Two staged spectral filtering

To minimize optical image distortion, to protect all following optical elements and to minimize the fluence on the filter for all wavelength that are contained in the plasma self-emission, the first spectral filter stage is placed directly behind the objective (refer Fig. 1). The filter consists of two different layers of Schott-glasses (GG-455 and BG-39), each with ${6}\,{\mathrm{mm}}$ thickness. They restrict the transmission of the imaging beamline to the spectral band between ${450}\,{\mathrm{nm}}$ and ${600}\,{\mathrm{nm}}$, as it is shown in Fig. 2a. The second stage of spectral filtering is applied directly in front of the camera. It consists of two spectrally narrow dielectric bandpass filters (FBH520-40 and FL514.5-10 by Thorlabs Inc.). Both are centered around the probe wavelength and give a combined optical density $\ge {10}$ for all other wavelength. The more narrow filter is stacked closer to the camera. In Fig. 2b the broadband spectral transmission characteristic of the colored glass based first filter stage is well separated from the more sharp spectral transmission characteristic of dielectric bandpass filters from the second filter stage (between ${500}\,{\mathrm{nm}}$ and ${540}\,{\mathrm{nm}}$). Dielectric bandpass filters yield a fixed transmission for all wavelength and stacking of filters can lead to multiple reflections of plasma self-emission between the filters and cause several spots of self-emission on the camera. Glass filters can in principle be stacked with different glass thicknesses and yield a very high spectral contrast ratio between the probe and pump laser wavelength. As presented here, the combination of the advantages of both results in a very efficient spectral filtering.

### Absolute timing in the experiment

The relative timing between shots can be determined over the beam arrival monitor (BAM)^25^. To shift the relative time delays between different shadowgrams to an absolute time delay with respect to the peak of the pump laser pulse, the ionization response of the target is utilized. The procedure can be done as follows: The peak intensity of the pump laser has to be reduced below $10^{15}\,\hbox {W/cm}^{2}$ by blocking most part of the high intensity beam via a ceramic screen before the focusing optics and transmitting just a small portion of the beam through a small hole onto the target. By continuously changing the pump-probe delay and capturing shadowgrams of the target together with the corresponding timing on the BAM, the earliest timing of the shadowgram with observed ionization can be found. It defines the arrival time of the pump laser peak.

### PIC simulation

The PIC cycle uses the Yee field solver, Esirkepov current deposition and Boris particle push. The simulation uses 18 particles per cell. The pump laser pulse in the PIC simulation is initialized with p-polarization, ${800}\,{\mathrm{nm}}$ wavelength and a Gaussian shape in all three dimensions. The pulse duration FWHM is set to ${30}\,\mathrm{fs}$, ${4.1}\,{\upmu }\mathrm{m}$ FWHM into the lateral dimensions and the normalized peak vector potential is $a_0 = {33}$. The overall box size is ${20}\,{\upmu }\mathrm{m}$ (target axis) $\times {40.6}\,{\upmu }\mathrm{m} \times {80}\,{\upmu }\mathrm{m}$ (laser propagation direction) and the laser wavelength is isotropically resolved by 24 cells. One time step duration in the simulation is ${0.064}\,\mathrm{fs}$ and absorbing boundary conditions are used. The simulation runs on 60 GPUs of the type NVIDIA^®^ V100 and the run time is 4.5h.

## Supplementary Information

Supplementary Information.

## Data Availability

The datasets generated during and/or analyzed during the current study are available from the corresponding author on reasonable request.
