# Supplementary material for: Off-harmonic optical probing of high intensity laser plasma expansion dynamics in solid density hydrogen jets
Source: Sci Rep. 2022 May 4;12:7287. doi: 10.1038/s41598-022-10797-6 (PMC9068928; doi:10.1038/s41598-022-10797-6)
Supplement: Supplementary file 1 — Supplementary Information. [file 41598_2022_10797_MOESM1_ESM.pdf]

Supplementary information: Off-harmonic optical probing of high intensity laser plasma expansion dynamics in solid density hydrogen jets

Constantin Bernert[1,2,*], Stefan Assenbaum[1,2], Florian-Emanuel Brack[1,2], Thomas E. Cowan[1,2], Chandra B. Curry[3,4], Marco Garten[1,2], Lennart Gaus[1,2], Maxence Gauthier[3], Sebastian Göde[5], Ilja Goethel[1,2], Siegfried H. Glenzer[3], Thomas Kluge[1], Stephan Kraft[1], Florian Kroll[1], Michael Kuntzsch[1], Josefine Metzkes-Ng[1], Markus Loeser[1], Lieselotte Obst-Huebl[1,6], Martin Rehwald[1,2], Hans-Peter Schlenvoigt[1], Christopher Schoenwaelder[3,7], Ulrich Schramm[1,2], Mathias Siebold[1], Franziska Treffert[3,8], Tim Ziegler[1,2], and Karl Zeil[1]

[1]Helmholtz-Zentrum Dresden - Rossendorf, 01328 Dresden, Germany
[2]Technische Universität Dresden, 01062 Dresden, Germany
[3]SLAC National Accelerator Laboratory, Menlo Park, California 94025, USA
[4]University of Alberta, Edmonton, Alberta T6G 1H9, Canada
[5]European XFEL GmbH, 22869 Schenefeld, Germany
[6]Present address: Lawrence Berkeley National Laboratory, Berkeley, California, 94720, USA
[7]Friedrich-Alexander Universität Erlangen-Nürnberg, 91054 Erlangen, Germany
[8]Technische Universität Darmstadt, 64289 Darmstadt, Germany
[*]c.bernert@hzdr.de

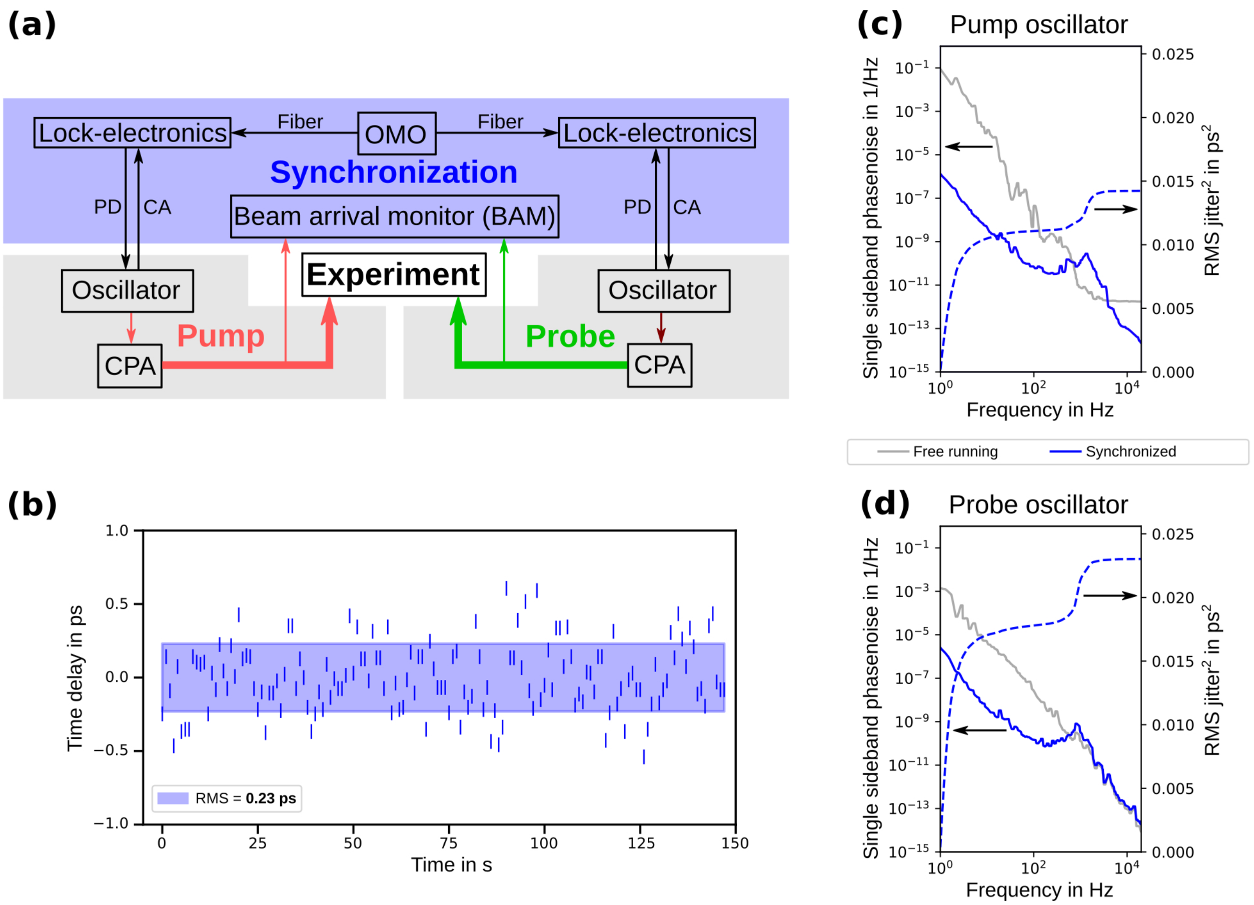

Figure S1. **(a)** Schematic of synchronization control between pump- and probe laser system, OMO - optical master oscillator, PD - photo diode, CA - cavity adjustment, CPA - chirped pulse amplification; **(b)** measurement of the shot-to-shot time delay via the beam arrival monitor (BAM), hight of the markers gives the error of the BAM measurement; **(c)**,**(d)** measurement of the single sideband phase noise versus an external clock and the calculated square of the temporal RMS-jitter for (c) pump laser oscillator and (d) probe laser oscillator.

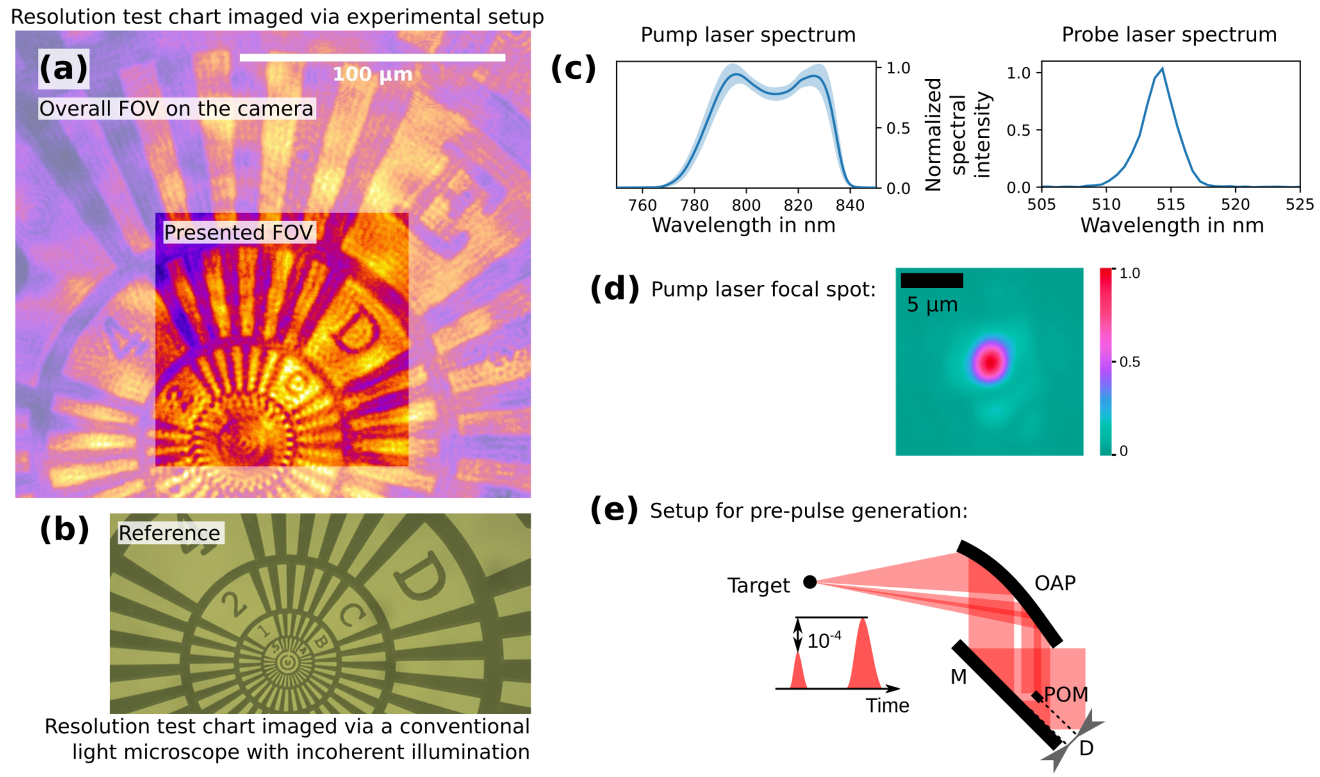

Figure S2. **(a)** Resolution test chart illuminated by the probe laser and imaged by the optical setup presented in figure 1 of the manuscript. The overall field of view (FOV) on the camera and the presented FOV (figure 3 in the manuscript) is shown. A structure size as small as 1.5 µm is resolved into all directions and no image distortion is visible within the overall FOV. **(b)** The same resolution test chart imaged by a conventional light microscope in bright-field operation and with incoherent illumination. The spatial scale is equivalent to figure (a). **(c) left:** Spectrum of the fully amplified pump laser beam before compression. The spectrum shows the mean and standard deviation over 37 days of laser operation. **(c) right:** Probe laser spectrum. Reproduction of the "Probe only" spectrum in figure 2 (c) of the manuscript. **(d)** Pump laser focal spot measured with the attenuated pump laser beam. Instead of the anti-reflection coating of the plasma mirror substrate, a reflective part of the same substrate is used to emulate the reflective plasma mirror surface during a high intensity laser shot. **(e)** Setup for generation of the −20 ps and −40 ps pre-pulse utilized for the measurements in figure 2 (c) of the manuscript. The pump laser beam with 18 cm beam diameter is reflected by the last folding mirror (M) to the off-axis parabola (OAP) and focused onto the target. A pick-off mirror (POM) with half-inch diameter is inserted before the last folding mirror and generates a pre-pulse with defined delay (D) at 10^{-4} less intensity than the peak of the pump laser pulse.
